# Closed-Eye Visualizations in the Setting of Hyponatremia

**DOI:** 10.1155/2018/5127917

**Published:** 2018-02-11

**Authors:** Travis Peck, Christopher Mercogliano, Eugene York

**Affiliations:** Department of Internal Medicine, Reading Health System, Reading, PA, USA

## Abstract

**Purpose:**

To report a case of closed-eye visualizations and to clarify the different types of hallucinations and their etiologies.

**Methods:**

Retrospective case report of a patient with closed-eye visualizations secondary to hyponatremia. Clinical findings, physical exam, laboratory assessment, treatment, and disease course from the patient's hospitalization were used in creating this report. Follow-up data after discharge were also obtained.

**Results:**

Closed-eye visualizations were diagnosed as secondary to hyponatremia, as they did not occur with the eyes open, and potential alternate causes were excluded. Serum sodium nadir was 119 mEq/L. Symptoms resolved with correction of hyponatremia via fluid resuscitation and electrolyte replenishment. There has been no recurrence of the symptoms.

**Conclusion:**

This patient had hallucinations exclusively with the eyes closed, which must be differentiated from the release hallucinations seen with the eyes open in Charles Bonnet syndrome. This patient had no visual loss or retinal disease, which should be suspected in open eye hallucinations.

## 1. Introduction

A hallucination is the perception of an object or an event in the absence of an external stimulus. Visual hallucinations can be categorized as simple—lights, colors, or shapes—or complex with objects and people [1]. Causes include psychosis, drugs, delirium, Charles Bonnet syndrome, compressive tumors, migraines, and hypnagogic phenomena [2]. A unifying feature of these hallucinations is that they occur when the patient's eyes are open. A review of the literature shows that cases of closed-eye hallucinations have been reported in postoperative patients who received general anesthesia or rarely in temporal lobe epilepsy [3]. The patient in this case had not received any drugs known to be hallucinogenic and did not have history of seizures or any evidence of seizure during hospitalization.

Hyponatremia can be defined as a serum sodium concentration less than 135 mEq/L. This is a known cause of neurologic symptoms, typically at levels below 120 mEq/L. Of patients with serum sodium less than 120 mEq/L, 0.5% report hallucinations [4]. However, this is the first reported case of visual hallucinations occurring exclusively with eye closure secondary to hyponatremia. It is critical to recognize these visualizations as a correctable symptom of an electrolyte abnormality.

## 2. Case Description

An 80-year-old male with hypertension and coronary artery disease presented with a three-day history of emesis and diarrhea, diagnosed as gastroenteritis. He endorsed dizziness and weakness but did not report any headache, blurry vision, paresthesias, or syncope. The patient also described a three-day history of complex visualizations involving a moving car. The car had color and appeared life-like. It was not distorted and seemed to be driving in a realistic manner in front of him. This occurred exclusive when his eyes were closed and immediately resolved upon opening his eyes. It was present for the majority of the time his eyes were closed and made it difficult for him to sleep.

He denied any prior hallucinations and had insight that the car was not real. He had no history of head trauma, cerebrovascular disease, or personal or familial history of psychosis or dementia. Ophthalmologic history was unremarkable and he used glasses only for reading. He was not experiencing any other sensory hallucinations or illusions at this time.

The patient was hemodynamically stable and afebrile, and physical examination was significant only for mild abdominal tenderness. There were no signs of cognitive impairment. Fundoscopic examination did not reveal any abnormalities. Laboratory analysis showed white blood cell count 4.6 billion cells/L, sodium 119 mEq/L, potassium 3.2 mEq/L, carbon dioxide 24.6 mEq/L, anion gap 10, glucose 186 mg/dL, and lactate 2.1 mmol/L. Urine drug screen was negative. Chest X-ray was normal, and electrocardiogram showed normal sinus rhythm.

Fluid resuscitation with normal saline and electrolyte replenishment was initiated. The visualizations decreased in frequency over the next two days and were completely absent by the third day after admission, which was the sixth day since they first began. Serum sodium increased during treatment and was 130 mEq/L on the day the hallucinations subsided. The patient was discharged the next day and has not experienced any recurrence of closed-eye visualizations or other hallucinations in the eight months since.

## 3. Discussion

Visual hallucinations are often the manifestation of underlying neurologic or ophthalmologic pathology. In these patients, it is critical to take a thorough history including whether the visualizations are simple or complex, specific content, presence of distortion, association with specific triggers, and whether the patient has insight into their reality. These details allow for the broad differential of hallucinations to be narrowed to specific causes.

The mechanism behind hallucinations in hyponatremia is unclear, but the etiology of visual hallucinations in general can be grouped into three categories: brain anatomy, brain chemistry, and emergence of the unconscious into the conscious [5]. Both brain anatomy and brain chemistry could be affected by electrolyte abnormalities. It is known that cerebral edema and intracellular swelling play a role in the central nervous system symptoms of hyponatremia [4]. The neurons in the visual cortex could also become more excitable through alterations in membrane potential. One of the leading theories regarding hallucinations is that they are due to an imbalance between inhibitory and excitatory influences on the brain [6]. It has been theorized that, in susceptible patients, the temporary visual deprivation of eye closure could be enough to cause spontaneous firing of the visual cortex due to lack of visual inputs [7].

This case illustrates the importance of determining whether visualizations are seen with eyes opened or closed. Visual release phenomena, such as those seen in Charles Bonnet syndrome, occur exclusively when the eyes are open. These patients most likely experience spontaneous firing of the visual cortex as a result of visual deprivation and deafferentation of visual cortical pathways [1]. There is typically no underlying psychiatric disease, and the patients are aware that what they are seeing are hallucinations [8]. The patient in this case report could have been diagnosed with this syndrome if it was not specifically elicited whether visualizations were present with eyes opened or closed, as the patient did not volunteer this information. It was crucial that this patient's symptoms were differentiated from Charles Bonnet syndrome, as this would have prompted an unnecessary ophthalmologic workup.

The symptoms experienced by the patient in this case are also phenomenologically similar to hypnagogic hallucinations. It is estimated that approximately 70% of the population experiences hallucinations at least once while transitioning from wakefulness to sleeping [9]. These are involuntary and typically complex, featuring bright colors and people or objects. The person experiencing these perceptions is fully aware that they are not real and rarely is affected emotionally by them [9]. This description fits the moving car visualized by our patient and his reaction to it. Hypnagogic hallucinations are thought to be due to increased cortical and thalamic activation, potentiated by acetylcholine in sleep, combined with spontaneous discharges resulting from cortical deafferentation [10]. It is possible that hyponatremia is another mechanism whereby the excitatory influences on the brain may become out of proportion to inhibitory influences.

This case documents a previously unreported symptom of hyponatremia, an exceedingly common problem in hospitalized patients. The correction of serum sodium is the only treatment necessary in cases of hallucinations, as this was sufficient to cure this patient's symptoms. Further workup and treatment can be avoided unless symptoms persist or there is evidence of alternate etiologies. Patients can also be reassured that the visualizations will be temporary.
